# Non-contrast cine cardiovascular magnetic resonance-based radiomics nomogram for predicting microvascular obstruction after reperfusion in ST-segment elevation myocardial infarction

**DOI:** 10.3389/fcvm.2023.1274267

**Published:** 2023-11-03

**Authors:** Xiaowen Liu, Ting Xu, Yongjia Peng, Jialin Yuan, Shuxing Wang, Wuyan Xu, Jingshan Gong

**Affiliations:** ^1^ The Second Clinical Medical College, Jinan University, Shenzhen, China; ^2^ Department of Radiology, Shenzhen People’s Hospital, The Second Clinical Medical College of Jinan University, The First Affiliated Hospital of Southern University of Science and Technology, Shenzhen, China; ^3^ Guangzhou Red Cross Hospital, Jinan University, Guangzhou, China

**Keywords:** non-contrast cine CMR, ST-segment elevation myocardial infarction, microvascular obstruction, radiomics, risk stratification

## Abstract

**Purpose:**

This study aimed to develop and validate a cine cardiovascular magnetic resonance (CMR)-based radiomics nomogram model for predicting microvascular obstruction (MVO) following reperfusion in patients with ST-segment elevation myocardial infarction (STEMI).

**Methods:**

In total, 167 consecutive STEMI patients were retrospectively enrolled. The patients were randomly divided into training and validation cohorts with a ratio of 7:3. All patients were diagnosed with myocardial infarction with or without MVO based on late gadolinium enhancement imaging. Radiomics features were extracted from the cine CMR end-diastolic volume phase of the entire left ventricular myocardium (3D volume). The least absolute shrinkage and selection operator (LASSO) regression was employed to select the features that were most relevant to the MVO; these features were then used to calculate the radiomics score (Rad-score). A combined model was developed based on independent risk factors screened using multivariate regression analysis and visualized using a nomogram. Performance was assessed using receiver operating characteristic curve, calibration curve, and decision curve analysis (DCA).

**Results:**

The univariate analysis of clinical features demonstrated that only cardiac troponin I (cTNI) was significantly associated with MVO. LASSO regression revealed that 12 radiomics features were strongly associated with MVO. Multivariate regression analysis indicated that cTNI and Rad-score were independent risk factors for MVO. The nomogram based on these two features achieved an area under the curve of 0.86 and 0.78 in the training and validation cohorts, respectively. Calibration curves and DCA indicated the clinical feasibility and utility of the nomogram.

**Conclusions:**

A CMR-based radiomics nomogram offers an effective means of predicting MVO without contrast agents and radiation, which could facilitate risk stratification of patients with STEMI after PCI for reperfusion.

## 1. Introduction

Percutaneous coronary intervention (PCI) is the preferred treatment for acute myocardial infarction (AMI). Promptly restoring blood flow through PCI in occluded arteries significantly reduces the mortality rate of patients with ST-segment elevation myocardial infarction (STEMI) (1). Despite successful epicardial coronary artery revascularization, approximately 40% of patients cannot attain optimal myocardial perfusion due to coronary microvascular dysfunction (CMD) following ischemia (2, 3). This condition is associated with a long-term risk of adverse outcomes (4–7). Microvascular obstruction (MVO) detected on cardiovascular magnetic resonance (CMR) is the most commonly used index for assessing CMD following STEMI (6, 8, 9). MVO is defined as a dark zone within the hyperintense myocardial infarction region observed on late gadolinium enhancement (LGE) images. Several studies have consistently confirmed the accuracy and reliability of cardiac MRI using LGE for non-invasive MVO assessment establishing it as the gold standard for visualizing MVO (10–12).

To complete LGE imaging, it is essential to administer an intravenous injection of gadolinium and conduct a delayed scan 15 min after contrast administration. This procedure not only is time-consuming and labor-intensive but also carries a risk of nephrogenic systemic fibrosis (NSF) in patients with severe renal disease. Given the frequent coexistence of kidney and cardiovascular diseases (CVDs), this matter has garnered significant attention in the clinical setting (13). Therefore, non-contrast CMR protocols for risk stratification in patients with STEMI should be implemented clinically. Recent research has indicated that non-contrast cine CMR images can serve as an alternative approach to LGE-CMR, enabling the diagnosis of CVDs without the need for gadolinium injection (14–16).

Radiomics is a quantitative image analysis method that transforms images into mineable data, allowing the extraction of detailed information on myocardial characteristics and thereby providing new information from existing standard images (17–19). Radiomics has great potential for transforming imaging data into clinically applicable models for disease diagnosis and prognostic assessment. In addition, radiomics can be used to evaluate treatment response or predict certain prognostic characteristics and is currently widely applied in the field of CVDs. Cine CMR, based on a balanced steady-state free precession (bSSFP) gradient echo sequence, is routinely implemented to assess heart function using CMR protocols. Owing to the bSSFP signal being the coherent sum of SSFP-free induction decay and SSFP-echo, it may bear both T2 and T2 star components, even though the T2 star component is usually negligible. Therefore, we hypothesize that radiomics could mine the features produced by the T2 star component of the bSSFP signal. This study aimed to develop and validate a cine CMR-based radiomics model to predict MVO in patients with STEMI after PCI.

## 2. Materials and methods

### 2.1. Patient population

This study was approved by the institutional review board. The requirement for informed consent was waived owing to the retrospective nature of the study. The inclusion criteria were as follows: (1) patients who underwent PCI within 12 h from onset of symptoms and a diagnosis of acute STEMI based on ST-segment elevation of >0.1 mV in at least two contiguous limb leads or >0.2 mV in the precordial leads, along with coronary angiography revealing more than 50% stenosis in two or more coronary arteries and successful restoration of patency to the occluded vessels, and (2) patients who underwent CMR examination within 2–6 days after PCI and had complete clinical data. The exclusion criteria were as follows: (1) patients with a history of prior myocardial infarction or previous PCI; (2) patients with other cardiomyopathies, severe valvular disease, acute pericarditis, myocarditis, or recent severe cardiac insufficiency; and (3) poor CMR image quality and inadequate image segmentation. Between January 2021 and April 2023, 167 patients were enrolled and randomly divided into training and validation cohorts with a ratio of 7:3. Relevant clinical data were collected from all patients.

### 2.2. CMR image acquisition

CMR examination was conducted using a 3.0T MR system (MAGNETOM Skyra; Siemens Healthcare) equipped with an 18-channel cardiac phased-control coil and facilitated with respiratory gating and ECG vector gating. True fast imaging with a steady-state precession (true FISP) sequence was employed to capture the standard left ventricular (LV) short axis and LV two-chamber and four-chamber cine CMR. The LV short-axis images cover the entire left ventricle. LV ejection fraction (LVEF %), end-systolic volume (ESV, ml), end-diastolic volume (EDV, ml), and LV mass (g) were assessed using cine imaging. The images were resampled to 1 mm  × 1 mm × 3 mm and normalized to 0–240 gray scale. LGE imaging was conducted 15 min after the intravenous administration of a gadolinium-based contrast agent at a dose of 0.1 mmol/kg, using a two-dimensional T1-weighted inversion recovery gradient echo sequence. Inversion times were individually selected from 250 to 400 ms to optimize the nulling of the unaffected myocardium. The scanning parameters are listed in Supplementary Material S1.

### 2.3. Image preprocessing and radiomics feature extraction

**Figure 1:**
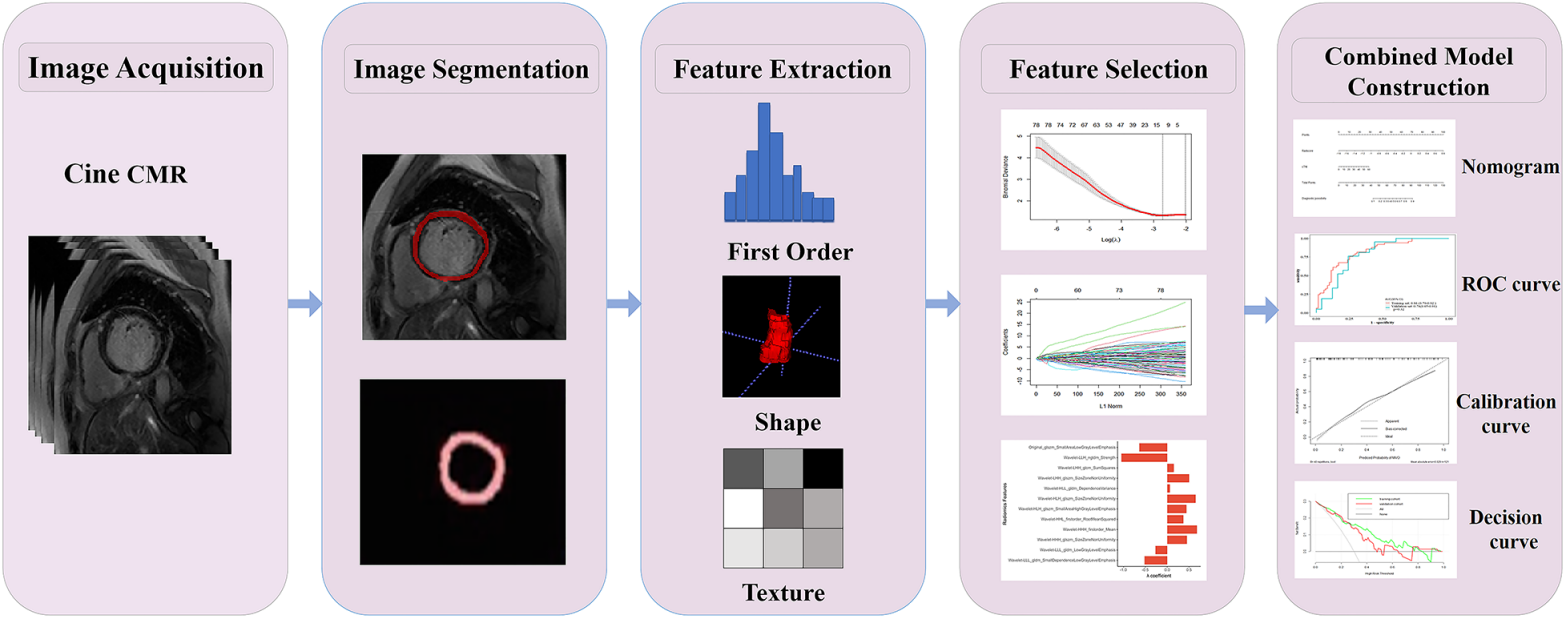
Radiomics flowchart of the current study. Layer-by-layer manual segmentation of the myocardium was performed using ITK-SNAP. The radiomics features, including first-order, shape, texture features, and others, were extracted using the PyRadiomics. For feature selection, the extracted features were chosen through interobserver and intraobserver reliability assessment and LASSO. The Rad-score of the radiation cohort was constructed from a linear combination of selected features. The predictive performance was assessed using the AUC of the ROC. To improve the interpretation of results, we developed a nomogram personalized assessment tool that evaluates the fitting excellence of column lines by calibrating curves and analyses the clinical utility of column lines by DCA curves.

Two experienced diagnostic radiologists (with more than 8 years of experience in diagnosing CMR images) interpreted the CMR images on the picture archiving and communication system (PACS) in consensus. LGE was defined as signal intensity exceeding five standard deviations above the mean signal intensity of the remote myocardium, serving as the gold standard for myocardial infarction evaluation (12). An MVO was defined as a low-signal area within the LGE high-signal myocardial infarction zone (20). Myocardium manual segmentation was carried out using the ITK-SNAP software (http://www.itksnap.org/pmwiki/pmwiki.php). The entire LV myocardium was delineated as the volume of interest (VOI) within the end-diastolic volume of the cine CMR images by two radiologists (with more than 5 years of experience in CMR imaging) manually, as depicted in Figure 1. On average, each myocardial segmentation took approximately 6.5 ± 0.75 min. In total, 167 VOIs were identified in 167 patients. The open-source software PyRadiomics package (https://pyradiomics.readthedocs.io) was used to extract the radiomics features (21). Image normalization is crucial to ensure the comparability of features among different cine CMR images. The N4 bias field correction algorithm was employed during the preprocessing step to address image inhomogeneity. Subsequently, feature extraction was conducted on the complete LV myocardium (3D volume) at the end diastole. A total of 854 radiomics features were extracted, which consisted of 18 first-order statistical features, 12 shape features, 24 gray-level co-occurrence matrix (GLCM) features, 16 gray-level run length matrix (GLRLM) features, 16 gray-level size zone matrix (GLSZM) features, 14 gray-level dependence matrix (GLDM) features, and five neighborhood gray-tone difference matrix (NGTDM) features. In addition, several image filters were applied to the original image to obtain corresponding derived images, including wavelet. The details pertaining to the radiomics feature extraction are shown in Supplementary Material S2.

### 2.4. Radiomics feature selection

The radiomics signature development pipeline was as follows. First, intraclass correlation coefficients (ICCs) were calculated to exclude features with low reliability (ICCs < 0.80), and the mean values of the included feature values of the segmentation of the radiologists were used for further analysis. Second, the data of all radiomics features were standardized with the *Z*-score, and Spearman's rank correlation coefficient was used to remove redundant features with strong correlations. For features with a correlation coefficient of >0.9, only one was retained while the other was dismissed. Third, the least absolute shrinkage and selection operator (LASSO) regression with fivefold cross-validation was implemented to select the strongest features for calculating the radiomics score (Rad-score) through a linear combination (Supplementary Material S3). A multivariate logistic regression model integrating the Rad-score and clinical features was developed and visualized using a nomogram. The workflow for the construction and validation of the radiomics model is shown in Figure 1.

### 2.5. Statistical analysis

Statistical analyses were performed using R (R Foundation for Statistical Computing) and SPSS version 22.0. Statistical significance was set at *p* < 0.05. Correlations between clinical data, imaging histological features, and MVO were analyzed using the *t*-test, chi-square test, or Mann–Whitney rank-sum test. Continuous variables were expressed as mean ± SD or median (lower and upper quartile), while categorical variables were reported as frequencies and percentages. Univariate and multivariate logistic regression analyses were conducted to identify the clinical factors that could independently predict the MVO status. All variables associated with MVO status at a significance level of *p* < 0.05 in the univariable analysis were included in the multivariable logistic regression analysis. Predictive performance was assessed using the area under the curve (AUC) of the receiver operating characteristic (ROC) curve and compared with the DeLong test. Furthermore, various diagnostic capabilities of the prediction models were assessed by implementing the sensitivity, specificity, positive predictive value (PPV), and negative predictive value (NPV). The calibration efficiency of the nomogram was assessed by drawing calibration curves. The decision curve analysis (DCA) was performed to evaluate the clinical utility of the predictive models.

## 3. Results

### 3.1. Study population

**Figure 2:**
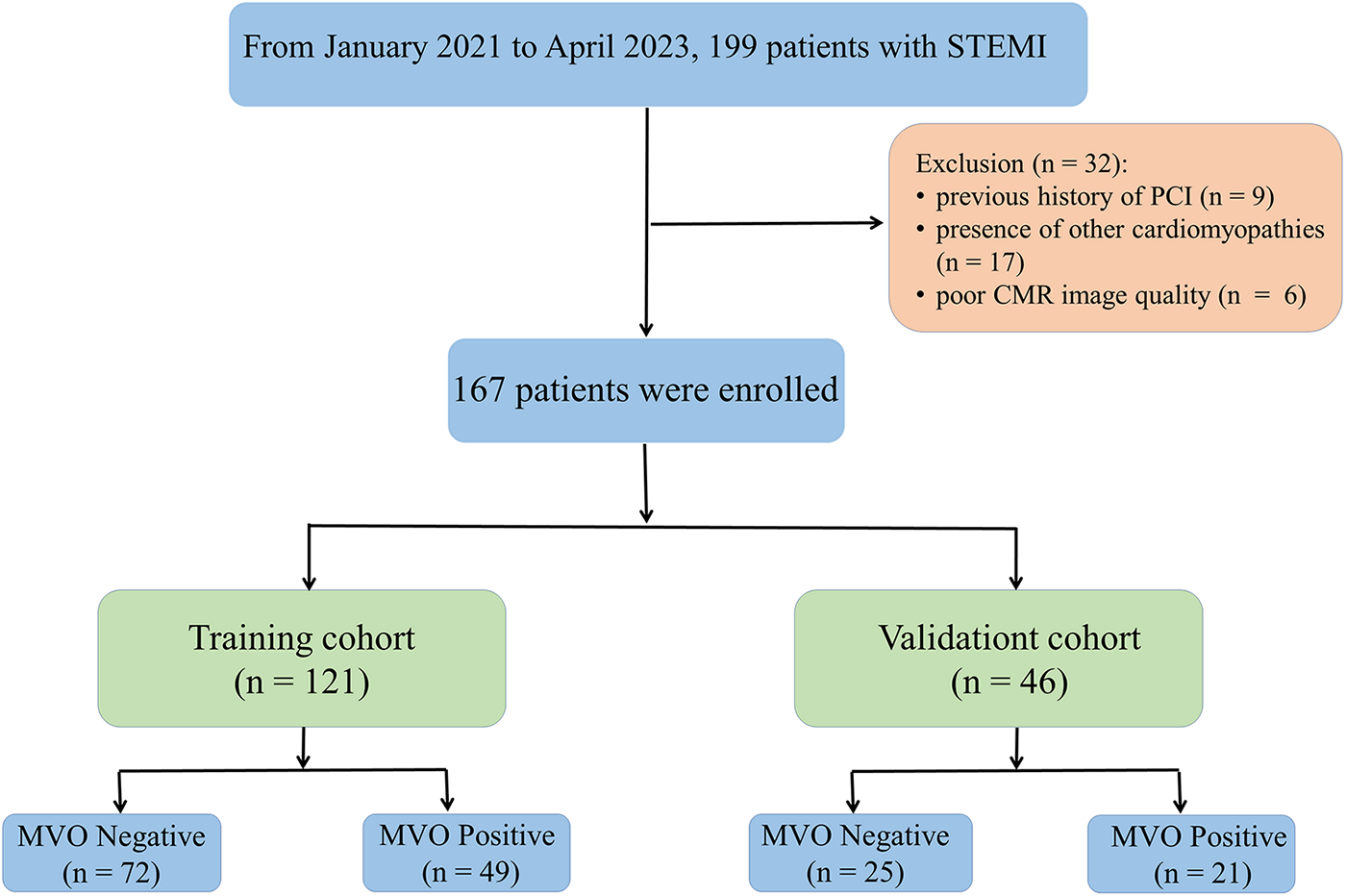
Flowchart of inclusion and exclusion criteria. A total of 167 patients were enrolled in this study and randomly divided into a training (*n* = 121) and validation cohort (*n* = 46) at a ratio of 7:3.

The demographic characteristics of the study population are presented in Table 1. A total of 32 patients were excluded because of a history of PCI (*n* = 9), presence of other cardiomyopathies (*n* = 17), and poor CMR image quality (*n* = 6). A total of 167 eligible patients were included in this study, consisting of 70 (41.91%) patients with MVO and 97 (58.09%) patients without MVO after PCI (Figure 2). In the training cohort, there were significant differences in infarction size, LVEF, ESV, EDV, heart rate (HR), cardiac troponin I (cTNI), and Rad-score between MVO-negative and MVO-positive patients. In the validation cohort, infarction size, left ventricular mass (LVM), cTNI, alcohol consumption, and Rad-score were significantly different between the MVO-negative and MVO-positive patients (*p* < 0.05). In addition, the Rad-score for patients with myocardial infarction with MVO was higher than that in patients without MVO.

**Table 1:** Participant characteristics.

Variable	Training cohort (*n* = 121)		*P*-value	Validation cohort (*n* = 46)		*P*-value
	MVO negative (*n* = 72)	MVO positive (*n* = 49)		MVO negative (*n* = 25)	MVO positive (*n* = 21)	
Age (years)	52.1 ± 12.5	50.5 ± 10.4	0.476	52.9 ± 11.4	54.4 ± 9.4	0.641
Gender			1			0.09
Male	64 (88.9)	44 (89.8)		20 (80)	21 (100)	
Female	8 (11.1)	5 (10.2)		5 (20)	NA	
Time to balloon (min)	180 (120–360)	300 (165–405)	0.119	180 (135–300)	240 (165–390)	0.534
CMR time (days)	3.8 ± 1.4	3.7 ± 1.6	0.623	3.4 ± 1.7	3.9 ± 1.5	0.262
Culprit vessel			0.762			0.09
LAD	46 (63.9)	33 (67.3)		19 (76)	11 (52.4)	
RCA	20 (27.8)	12 (24.5)		5 (20)	7 (33.3)	
LCX	6 (8.3)	4 (8.2)		1 (4)	3 (14.3)	
Infarction size (%)	16.1 ± 1.73	28.9 ± 3.1	<0.001	16.1 ± 2.4	27.9 ± 2.7	<0.001
LVEF (%)	56 (46.5–61)	46 (38–56.5)	<0.001	56 (45.5–62)	48 (37.5–54)	0.15
ESV (ml)	62 (45.3–79.8)	83 (65.5–115)	0.019	68 (51–103.5)	88 (63.5–135.5)	0.193
EDV (ml)	142.5 (124.8–169.3)	168 (136.5–190)	0.0031	155 (124.5–182)	173 (131–238)	0.183
CO (L/min)	5 (4.6–5.7)	5.2 (4.3–5.7)	0.859	4.7 (4.5–6.4)	5.1 (4.7–6.2)	0.514
HR (bpm)	66.5 (59–75)	71 (63.5–79)	0.043	65 (55.5–72.5)	66 (61–80)	0.119
LVM (g)	142.6 (127.3–168.9)	155.3 (133.8–180.9)	0.805	153.3 (127.7–172.6)	166 (137.2–189.2)	0.033
cTNI (μg/L)	2.6 (0.13–11.7)	15.4 (6.2–37.4)	<0.001	3.7 (0.6–8.9)	13.2 (3.5–32.7)	0.043
NT-ProBNP (pg/ml)	502.5 (264.5–1,177.8)	904 (297.2–1,786.2)	0.211	666 (286.5–1,559)	925 (187–1,738)	0.421
MPV (fl)	10.3 ± 0.9	10.5 ± 1.1	0.192	10.4 ± 0.8	10.5 ± 0.9	0.49
LDL (mmol/L)	2.9 (2.3–3.6)	2.6 (1.9–3.3)	0.125	2.5 (1.8–3.8)	2.6 (1.9–3.4)	0.614
TG (mmol/L)	1.5 (1.1–2.4)	1.48 (1.1–2.6)	0.614	1.8 (0.9–1.9)	1.3 (1.1–1.7)	0.967
BUN (mmol/L)	5.2 (4.1–6.4)	4.7 (3.8–5.7)	0.755	4.9 (4.1–7.3)	5.4 (3.8–7.1)	0.855
Cre (μmol/L)	83.5 (74–97.5)	81 (73.6–89.5)	0.228	84 (68–95)	79.9 (70.4, 102.5)	0.372
Smoking			0.378			1
No	35 (48.6)	19 (38.8)		7 (28.0)	6 (28.6)	
Yes	37 (51.4)	30 (61.2)		18 (72.0)	15 (71.4)	
Alcohol consumption			0.494			0.046
Never	49 (68.1)	37 (75.5)		21 (84.0)	11 (52.4)	
Current/former	23 (31.9)	12 (24.5)		4 (16.0)	10 (47.6)	
Rad-score	−0.60 ± 0.45	−0.12 ± 0.30	<0.001	−1.59 ± 0.67	−0.92 ± 0.43	<0.001

LAD, left anterior descending (coronary artery); RCA, right coronary artery; LCX, left circumflex; CO, cardiac output; cTnI, cardiac troponin I; NT-ProBNP, N-terminal pro-brain natriuretic peptide; MPV, mean platelet volume; LDL, low-density lipoprotein; TG, triglyceride; BUN, blood urea nitrogen; Cre, creatinine.

### 3.2. Rad-score and the integrated model construction

**Figure 3:**
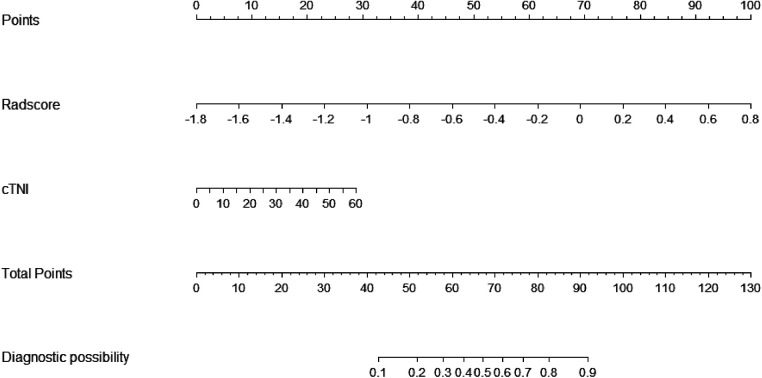
Nomogram based on the combined model of cTNI and Rad-score. The probability of each predictor (cTNI, Rad-score) can be converted into a risk score according to the “points” at the top of the nomogram. After calculating the total points by adding up these predictors, the corresponding prediction risk probability of MVO is at the bottom of the radiomics nomogram.

**Figure 4:**
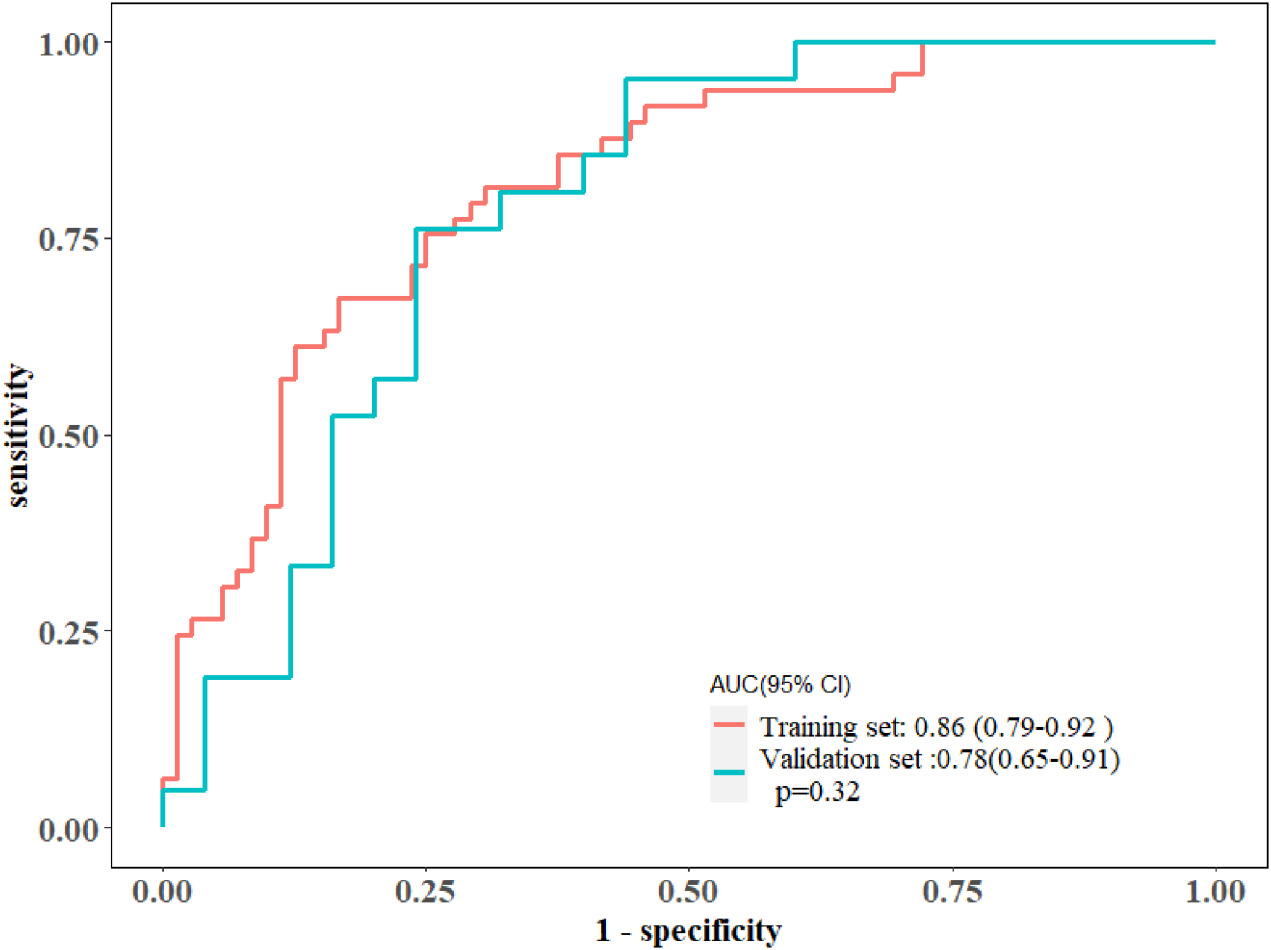
The ROC curves of the radiomics nomogram in the training and validation cohorts, along with their corresponding 95% confidence intervals.

**Figure 5:**
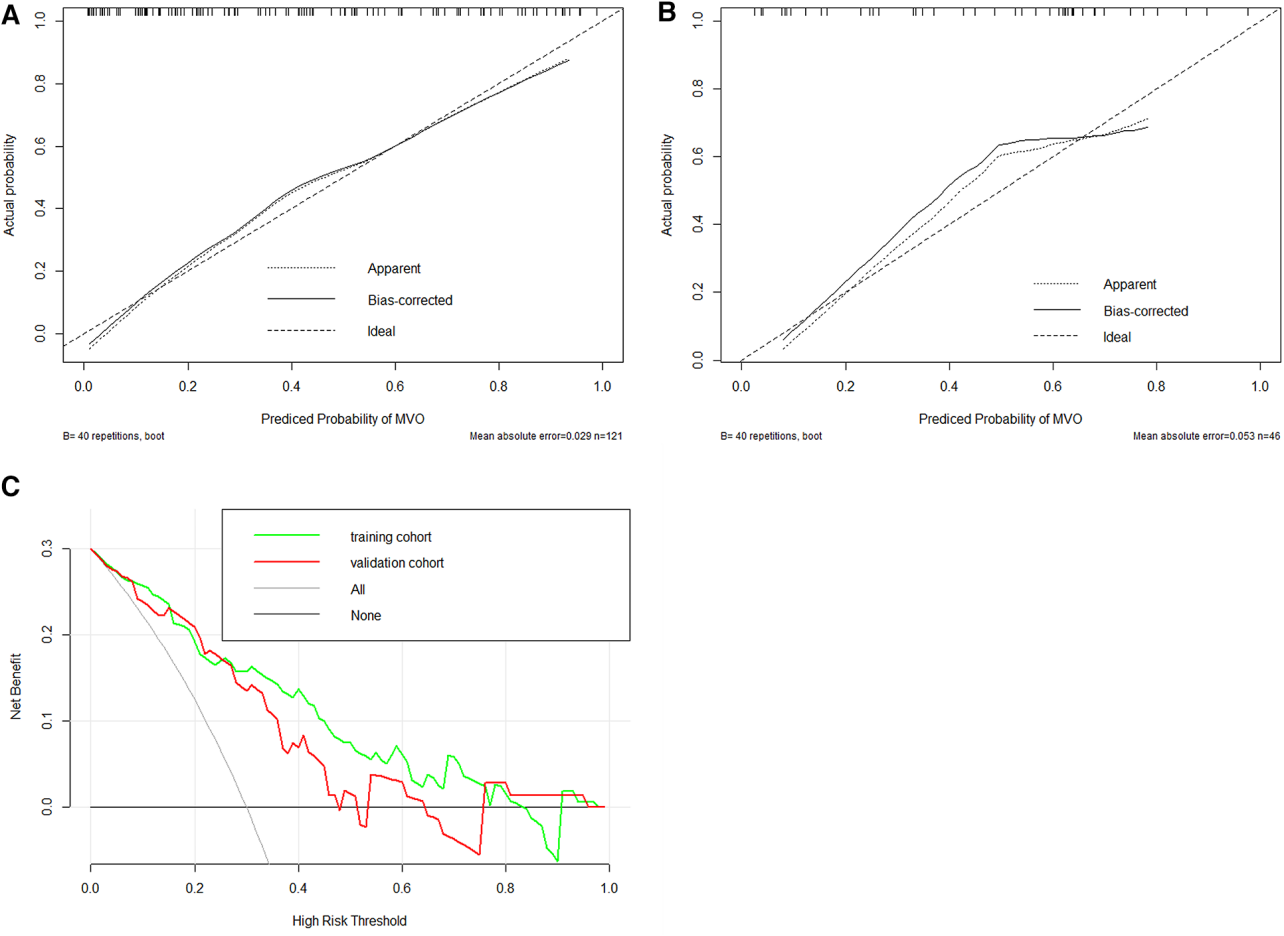
Evaluation of calibration and clinical utility of a radiomics nomogram in the training (**A**) and validation (**B**) cohorts. The calibration curves of the radiomics nomogram in the two cohorts are close to the ideal line, indicating good agreement on the presence of MVO between the risk estimation by the nomogram and confirmation of LGE. The DCA curves of the radiomics nomogram in the training cohort (green line) and validation cohort (red line) (**C**). The *x*-axis means the threshold probability, and the *y*-axis shows the model benefit. The higher curve at any given threshold probability is the optimal prediction to maximize net benefit. The combined model achieved a great net benefit at most probability thresholds in the training and validation cohorts.

**Figure 6:**
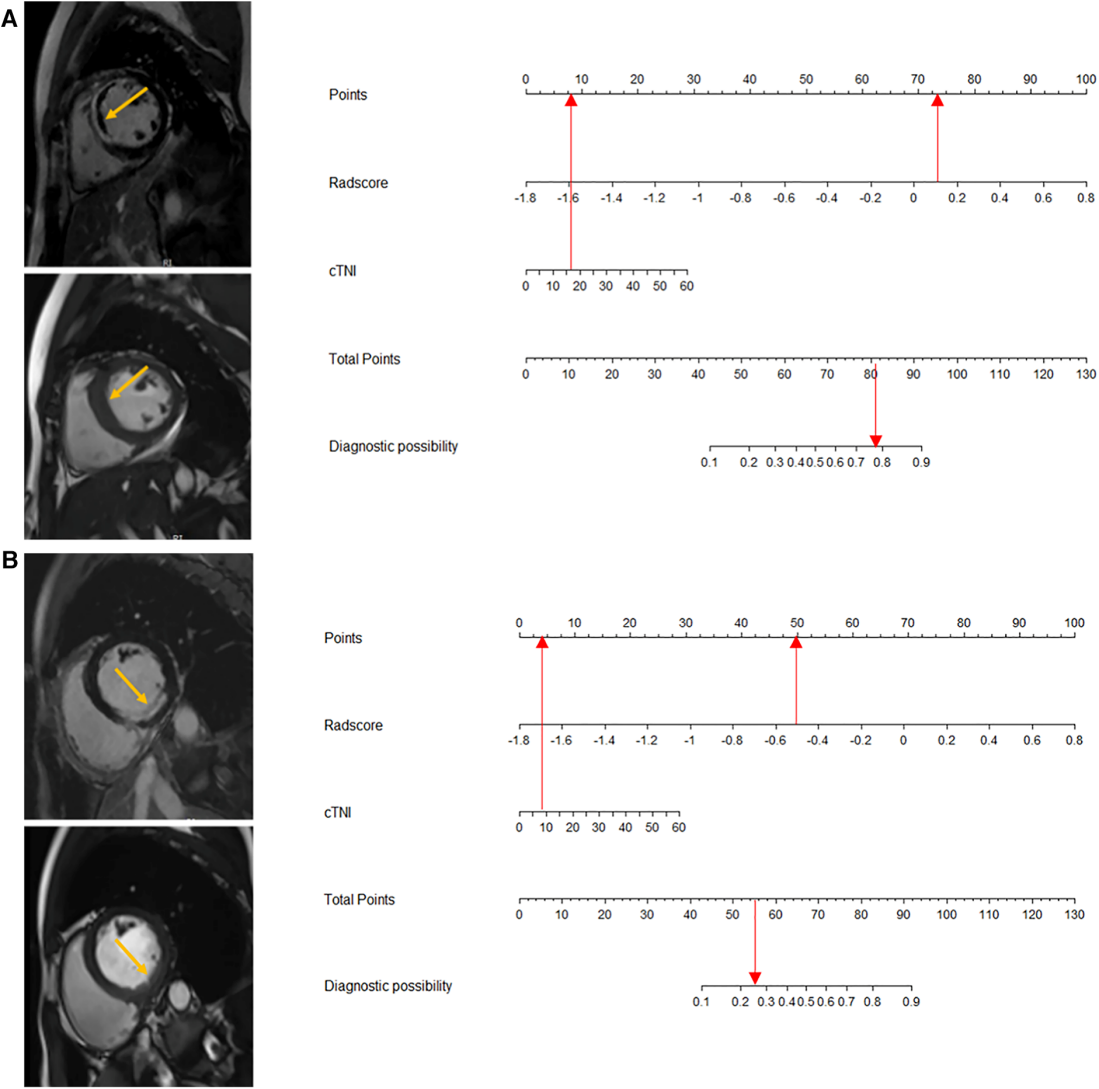
A 48-year-old male patient with positive MVO. The maximum section of the LGE lesion corresponds to the short-axis map of cine (yellow arrows). The cTNI level of the patient was 15.4 μg/L, which corresponds to a point score of 7.5. The Rad-score was 0.16, resulting in a corresponding point score of 73.5. The total point score was 81, and MVO risk exceeded 0.78 (**A**). A 65-year-old male patient with negative MVO. The maximum section of the LGE lesion corresponds to the short-axis map of cine (yellow arrows). The cTNI level of the patient was 9.53 μg/L, which corresponds to a point score of 4.8. The Rad-score was −0.5, resulting in a corresponding point score of 50. So the total point score was 54.8, and the risk of MVO was less than 0.25 (**B**).

In total, 559 radiomics features with ICCs > 0.80 were introduced into LASSO regression. Among them, 12 strongly correlated with MVO and were selected to calculate the Rad-score (Table 2) (Supplementary Methods S4). The AUCs of the Rad-score for MVO were 0.82 [95% confidence interval (CI): 0.74–0.89] and 0.78 (95% CI: 0.65–0.92) in training and validation cohorts, respectively, and the difference was not statistically significant. The multivariate logistic regression revealed that cTNI [odds ratio (OR) 1.04, 95% CI: 1.01–1.08] and Rad-score (OR 31.29, 95% CI: 7.58–173.52) were independent risk factors for MVO in patients with STEMI after PCI (Table 3). The final nomogram model was obtained by integrating the clinical signatures with the Rad-score (Figure 3). The nomogram obtained predictive performance for MVO with AUCs of 0.86 (95% CI: 0.79–0.92) and 0.78 (95% CI: 0.65–0.91) in the training cohort and validation cohort, respectively (Figure 4). The sensitivity and specificity in the validation cohort were 0.86 and 0.7, respectively (Table 4). The DeLong test demonstrated that the model did not exhibit significant differences between the training and validation cohorts (*p* = 0.32), suggesting that it possesses a high capacity for generalization. The calibration curves for the nomogram demonstrated good agreement regarding the presence of MVO between the risk estimation by the nomogram and the confirmation of LGE in the training (Figure 5A) and validation (Figure 5B) cohorts. The result of DCA shows that using the nomogram for MVO prediction has more benefits than two extreme conditions [the predict-all-patient scheme (gray curve) and the predict-none scheme (horizontal black line)]. A larger area under the decision curve suggested a better clinical utility. The combined model achieved a high net benefit at most probability thresholds, indicating that the MVO prediction model could facilitate decision-making in clinical settings (Figure 5C). Representative cases are shown in Figure 6.

**Table 2:** Twelve statistically significant radiomics features selected by analysis.

Radiomics features	*λ* coefficient
original_glszm_SmallAreaLowGrayLevelEmphasis	−0.64
wavelet-LLH_ngtdm_Strength	−1.05
wavelet-LHH_glcm_SumSquares	0.15
wavelet-LHH_glszm_SizeZoneNonUniformity	0.50
wavelet-HLL_gldm_DependenceVariance	0.06
wavelet-HLH_glszm_SizeZoneNonUniformity	0.65
wavelet-HLH_glszm_SmallAreaHighGrayLevelEmphasis	0.44
wavelet-HHL_firstorder_RootMeanSquared	0.37
wavelet-HHH_firstorder_Mean	0.68
wavelet-HHH_glszm_SizeZoneNonUniformity	0.45
wavelet-LLL_gldm_LowGrayLevelEmphasis	−0.27
wavelet-LLL_gldm_SmallDependenceLowGrayLevelEmphasis	−0.52

LLH, LHH, HLL, HLH, HHL, HHH, and LLL, wherein L and H are low-pass (i.e., scaling) and high-pass (i.e., wavelet) functions, respectively; glszm, gray-level size zone matrix; glcm, gray-level co-occurrence matrix; gldm, gray-level dependence matrix; ngtdm, neighborhood gray-tone difference matrix.

**Table 3:** The univariate and multivariate logistic regression of MVO status based on clinical characteristics and Rad-score in the training set.

	Univariate logistic regression			Multivariate logistic regression		
Variables	OR value	95% CI	*P*-value	OR value	95% CI	*P*-value
LVEF	0.94	0.83–1.06	0.33			
ESV	1.00	0.95–1.03	0.97			
EDV	0.99	0.95–1.04	0.53			
CO	1.50	0.54–4.15	0.42			
HR	1.01	0.94–1.09	0.77			
LVM	1.00	0.98–1.01	0.58			
NT-ProBNP	1.00	0.99–1.00	0.90			
cTNI	1.04	1.01–1.08	0.02	1.04	1.01–1.08	0.005
MPV	1.18	0.65–2.17	0.59			
LDL	0.64	0.37–1.61	0.09			
TG	1.09	0.78–1.61	0.63			
BUN	1.04	0.71–1.55	0.84			
Cre	0.99	0.95–1.01	0.68			
Gender	0.57	0.054–4.52	0.62			
Age	1.02	0.98–1.08	0.36			
Drink	0.58	0.15–1.98	0.40			
Smoke	1.05	0.35–1.82	0.91			
Heartache time	1.01	0.98–1.04	0.34			
Rad-score	46.04	7.42–449.5	<0.001	31.29	7.58–173.52	<0.001

**Table 4:** ROC analysis of the integrated models.

Performance indicators	Nomogram model	
	Training cohort	Validation cohort
AUC (95% CI)	0.86 (0.79–0.92)	0.78 (0.65–0.91)
Sensitivity	0.78	0.86
Specificity	0.79	0.7
NPV	0.84	0.85
PPV	0.72	0.7

## 4. Discussion

In this study, we investigated the capability of non-contrast cine CMR in MVO diagnosis using radiomics analysis and machine learning algorithms. To the best of our knowledge, this is the first study to extract radiomics features from cine CMR images to predict MVO in patients with STEMI after PCI. The present study demonstrated that a nomogram integrating non-contrast cine CMR-based radiomics signature and cTNI could achieve high predictive accuracy for MVO in the training and validation cohort, which could provide a gadolinium-free CMR potential approach for risk stratification after reperfusion in patients with STEMI. The optimal cutoff value of the total nomogram score of 28.50, derived from the training cohort, obtained sensitivity, specificity, NPV, and PPV of 0.86, 0.70, 0.85, and 0.70 in the validation cohort, respectively.

Given that up to 40% of STEMI patients develop CMD despite prompt recanalization of the culprit epicardial coronary artery and CMD is associated with long-term adverse outcomes, early risk stratification becomes imperative for effective patient management. Although CMD can be assessed using invasive modalities, an increasing number of studies have shown that CMR findings such as MVO or intramyocardial hemorrhage (IMH) could achieve similar predictive performance for adverse outcomes in patients with STEMI after PCI (6). As the diagnosis of MVO requires an intravenous gadolinium-based contrast agent and delayed scanning, it can be time-consuming and has some limitations in patient selection due to the side effects of the contrast agent. Therefore, there is a need for gadolinium-free CMR protocols. Most studies have highlighted T2 star imaging of IMH as a surrogate imaging marker for MVO. They found that IMH was more closely associated with adverse outcomes than MVO (22). As T2 star imaging is not widely available in clinical settings, some studies have focused on other non-contrast CMR images. Bustin et al. (23) utilized a T2-prepared bSSFP sequence to obtain 3D-T2 mapping of the myocardium, revealing a notable correlation between areas exhibiting increased T2 values in patients with myocarditis and the presence of LGE. Our previous study showed that radiomics features derived from cine CMR images were associated with MVO (24). Based on the results of this study, we attempted to establish and validate a nomogram model for predicting MVO in patients with STEMI. Cine CMR imaging, conducted using a bSSFP or true FISP sequence, harbors both T2 and T2 star components. Therefore, it has the potential to mirror prolonged T2 relaxation due to edema in myocardial infarction and reflect susceptibility to IMH. Unfortunately, the T2 star components of the bSSFP might be subtle and challenging for human visual interpretation. Radiomics, on the other hand, can capture the features induced by IMH through a comprehensive set of quantitative features.

In this study, the analysis of radiomics features extracted from non-contrast cine CMR revealed that 12 features had a strong correlation with MVO. The Rad-score, derived from these 12 features, exhibited significant differences between all patients who were diagnosed with myocardial infarction, with or without concurrent MVO, underscoring the ability of non-contrast cine CMR-based radiomics to reveal features that may not be apparent to the naked eye. This study represents the first effort to predict MVO in patients with STEMI after PCI using a non-contrast cine CMR-based radiomics nomogram. Several previous studies have demonstrated the value of cine CMR imaging in predicting the diagnosis of CVDs based on histological features. For instance, Avard et al. (25) reported that radiomics analysis of non-contrast cine CMR images achieved high accuracy in detecting myocardial infarction, offering a potential alternative diagnostic approach to LGE-CMR. Zhang et al. (26) developed a deep learning framework based on non-contrast cine CMR that could detect the presence, location, transmurality, and size of chronic myocardial infarction without relying on additional LGE images, demonstrating the diagnostic capabilities of routinely acquired non-contrast cine CMR images for chronic myocardial infarction diagnosis. Gräni et al. (27) included 48 STEMI patients who underwent CMR after PCI. Their study showed that segmental strain in patients with STEMI had a good diagnostic performance in identifying MVO+ and excellent diagnostic performance for LGE+ segments. It illustrated that segmental strain could serve as a potential contrast-free surrogate marker to enhance early risk stratification in patients after primary PCI. Recently, texture analysis (TA) has been explored for detecting subacute and chronic myocardial infarction using non-contrast cine CMR. Larroza et al. (28) found that TA based on cine CMR and LGE-CMR images could be used to differentiate AMI from chronic myocardial infarction with good diagnostic efficacy. Baessler et al. (15) employed TA to assess and discriminate between acute and chronic myocardial infarction tissues. Their objective was to showcase the diagnostic capabilities of non-contrast cine CMR images in myocardial infarction diagnosis. From the initially extracted 286 features using the random forest algorithm, five relevant features were identified and selected. Using logistic regression, they achieved an AUC of 0.93 for cine CMR, highlighting its strong efficacy in discriminating myocardial infarction tissues. Similarly, Schofield et al. (29) used cine CMR to differentiate the etiologies of LV hypertrophy (LVH) in multiple patients, aiming to reduce the reliance on gadolinium injection in CVDs. Their research suggested that the radiomics features extracted from bSSFP CMR datasets using TA held promise for distinguishing the etiologies of LVH. In this study, we aimed to develop and validate a cine CMR-based radiomics nomogram model, exploring the potential of radiomics. The utilization of non-contrast cine CMR radiomics could potentially reduce the need for gadolinium injection while enhancing the accuracy of MVO detection from CMR images. The combined model we developed demonstrated high predictive efficacy for MVO.

This study has some limitations. First, the retrospective study was conducted at a single center, resulting in a small sample size and images collected solely from one center. Consequently, the generalizability and stability of the findings may be limited. Future studies with larger sample sizes and multicenter designs are required to assess the efficacy of our constructed model and improve the robustness of the model. Second, our segmentation relied solely on one cardiac cycle (end diastole) and was manually segmented by cardiologists, which is a time-consuming and labor-intensive approach. With the gradual improvement in automatic cardiac segmentation techniques, we will attempt to reduce the workload by implementing automatic segmentation. Finally, our study did not utilize advanced technologies such as deep learning methods, which are end-to-end processes that bypass the need for segmentation, feature extraction, and data analysis. In future endeavors, we aim to harness these advanced algorithms to bolster the accuracy and predictive capabilities of our models.

## 5. Conclusions

In this study, we established a nomogram that can accurately predict the occurrence of MVO after reperfusion in patients with STEMI. This gadolinium-free approach for detecting MVO may facilitate risk stratification in patients with STEMI post-PCI. This represents a significant step toward personalized medicine for the management of patients with STEMI and has the potential to improve patient outcomes and reduce healthcare costs.

## Data Availability

The original contributions presented in the study are included in the article/**Supplementary Material**, further inquiries can be directed to the corresponding author.
