# Supplementary material for: Non-contrast cine cardiovascular magnetic resonance-based radiomics nomogram for predicting microvascular obstruction after reperfusion in ST-segment elevation myocardial infarction
Source: Front Cardiovasc Med. 2023 Nov 3;10:1274267. doi: 10.3389/fcvm.2023.1274267 (PMC10655024; doi:10.3389/fcvm.2023.1274267)
Supplement: Supplementary file 2 [file Table2.docx]

**Supplementary Materials**

**Supplementary Methods 1.** Magnetic resonance image acquisition parameters used in the present study.

| Parameters | Cine LGE | | |
| --- | --- | --- | --- |
| Acquisition plane | Short axis | Short axis |  | |
| TR (msec) | 39.2 | 700 |  | |
| TE (msec) | 1.4 | 2.0 |  | |
| Slice thickness (mm) | 8 | 8 |  | |
| Slice spacing (mm) | 1.6 | 1.6 |  | |
| FOV (mm) | 340×340 | 340×340 |  | |
| Matrix | 208×200 | 256×240 |  | |
| Flip angle (°) | 80 | 20 |  | |
| Contrast agent acquisition time (min) | - | 10-15 |  | |
| Dose of LGE (mmol/kg) | - | 0.1 |  | |

**Supplementary Methods 2.**

**Radiomics Feature Extraction**

This study extracted the radiomic features of Cine-MRI images using the PyRadiomics package [1]. A total of 854 radiomics features were extracted, which consisted 18 first-order statistical features, 12 shape features, 24 Gray Level Co-occurrence Matrix (GLCM) features, 16 Gray Level Run Length Matrix (GLRLM) features, 16 Gray Level Size Zone Matrix (GLSZM) features, 14 Gray Level Dependence Matrix (GLDM) features, and 5 Neighborhood Gray Tone Difference Matrix (NGTDM) features. In addition, several image filters were applied to original image to obtain corresponding derived images, including Wavelet.

**Radiomics Feature description**

Table 1. Feature categories used in this study.

| **Feature classes** | **Representative features** |
| --- | --- |
| **First order** | Energy, Total Energy, Entropy, Minimum, 10^th^ percentile, 90^th^ percentile, Maximum, Mean, Median, Interquartile Range, Range, Mean Absolute Deviation (MAD), Robust Mean Absolute Deviation (rMAD), Root Mean Squared (RMS), Skewness, Kurtosis, Variance, Uniformity. |
| **GLCM** | Autocorrelation, Joint Average, Cluster Prominence, Cluster Shade, Cluster Tendency, Contrast, Correlation, Difference Average, Difference Entropy, Difference Variance, Joint Energy, Joint Entropy, Informational Measure of Correlation (IMC) 1, Informational Measure of Correlation (IMC) 2, Inverse Difference Moment (IDM), Maximal Correlation Coefficient (MCC), Inverse Difference Moment Normalized (IDMN), Inverse Difference (ID), Inverse Difference Normalized (IDN), Inverse Variance, Maximum Probability, Sum Average, Sum Entropy, Sum of Squares. |
| **GLRLM** | Short Run Emphasis (SRE), Long Run Emphasis (LRE), Gray Level Non-Uniformity (GLN), Gray Level Non-Uniformity Normalized (GLNN), Run Length Non-Uniformity (RLN), Run Length Non-Uniformity Normalized (RLNN), Run Percentage (RP), Gray Level Variance (GLV), Run Variance (RV), Run Entropy (RE), Low Gray Level Run Emphasis (LGLRE), High Gray Level Run Emphasis (HGLRE), Short Run Low Gray Level Emphasis (SRLGLE), Short Run High Gray Level Emphasis (SRHGLE), Long Run Low Gray Level Emphasis (LRLGLE), Long Run High Gray Level Emphasis (LRHGLE). |
| **GLSZM** | Small Area Emphasis (SAE), Large Area Emphasis (LAE), Gray Level Non-Uniformity (GLN), Gray Level Non-Uniformity Normalized (GLNN), Size-Zone Non-Uniformity (SZN), Size-Zone Non-Uniformity Normalized (SZNN), Size-Zone Non-Uniformity Normalized (SZNN), Size-Zone Non-Uniformity Normalized (SZNN), Zone Variance (ZV), Zone Entropy (ZE), Low Gray Level Zone Emphasis (LGLZE), High Gray Level Zone Emphasis (HGLZE), Small Area Low Gray Level Emphasis (SALGLE), Small Area High Gray Level Emphasis (SAHGLE), Large Area Low Gray Level Emphasis (LALGLE), Large Area High Gray Level Emphasis (LAHGLE) |
| **GLDM** | Small Dependence Emphasis (SDE), Large Dependence Emphasis (LDE), **Gray Level Non-Uniformity (GLN),** Dependence Non-Uniformity (DN), Dependence Non-Uniformity Normalized (DNN), Gray Level Variance (GLV), Dependence Variance (DV), Dependence Entropy (DE), Low Gray Level Emphasis (LGLE), High Gray Level Emphasis (HGLE), Small Dependence Low Gray Level Emphasis (SDLGLE), Small Dependence High Gray Level Emphasis (SDHGLE), Large Dependence Low Gray Level Emphasis (LDLGLE), Large Dependence High Gray Level Emphasis (LDHGLE). |
| **NGTDM** | Coarseness, Contrast, Complexity, Busyness, Strength. |
| **Shape** | Elongation, MajorAxisLength, Maximum2DDiameterColumn, Maximum2DDiameterRow, Maximum2DDiameterSlice, Maximum3DDiameter, MeshVolume, MinorAxisLength, Sphericity, SurfaceArea, SurfaceVolumeRatio, VoxelVolume. |
| **Wavelet** | LLH_gldm_DependenceVariance, LLH_gldm_HighGrayLevelEmphasis… |

GLCM: Gray Level Co-occurrence Matrix, GLRLM: Gray Level Run Length Matrix, GLSZM: Gray Level Size Zone Matrix, GLDM: Gray Level Dependence Matrix, NGTDM: Neighboring Gray Tone Difference Matrix.

**Definition of the extracted radiomics features**

*First-order statistical features*

First-order statistics describe the distribution of voxel intensities within the image region defined by the mask through commonly used and basic metrics.

Let:

- X be a set of $N_{P}$ voxels included in the ROI
- P$\left( i \right)$ be the first order histogram with $N_{g}$ discrete intensity levels, where $N_{g}$ is the number of non-zero bins, equally spaced from 0 with a width defined in the binWidth parameter.
- $p\left( i \right)$be the normalized first order histogram and equal to $\frac{P\left( i \right)}{N_{p}}$

The following first-order statistical features were extracted:

1. **Energy**

$\text{energy}=\sum_{i=1}^{N_{p}} \left( X(i)+c \right)^{2}$

1. **Total Energy**

$\text{total energy}=V_{voxel}\sum_{i=1}^{N_{p}} \left( X(i)+c \right)^{2}$

1. **Entropy**

1. **Skewness**

$\mathrm{skewness}=\frac{\mu_{3}}{\sigma^{3}}=\frac{\frac{1}{N_{p}}\sum_{i=1}^{N_{p}} \left( X(i)-\bar{X} \right)^{3}}{\left( \sqrt{\frac{1}{N_{p}}\sum_{i=1}^{N_{p}} \left( X(i)-\bar{X} \right)^{2}} \right)^{3}}$

1. **Kurtosis**

$$\text{kurtosis}=\frac{\mu_{4}}{\sigma^{4}}=\frac{\frac{1}{N_{p}}\sum_{i=1}^{N_{p}} \left( X(i)-\bar{X} \right)^{4}}{\left( \frac{1}{N_{p}}\sum_{i=1}^{N_{p}} \left( X(i)-\bar{X} \right)^{2} \right)^{2}}$$

2. **90^th^ Percentile**

**The 90^th^ percentile of X**

1. **10^th^ Percentile**

**The 10^th^ percentile of X**

1. **Mean**

$\mathrm{mean}=\frac{1}{N_{p}}\sum_{i=1}^{N_{p}} X(i)$

1. **Minimum**

**minimum = min (X)**

1. **Maximum**

**maximum = max (X)**

1. **Range**

Range = max (X) – min (X)

1. **Mean Absolute Deviation (MAD)**

$\mathrm{MAD}=\frac{1}{N_{p}}\sum_{i=1}^{N_{p}} \mid X(i)-\bar{X}\mid$

1. **Robust Mean Absolute Deviation (rMAD)**

$\mathrm{rMAD}=\frac{1}{N_{10-90}}\sum_{i=1}^{N_{10-90}} \mid X_{10-90}(i)-\bar{X}_{10-90}\mid$

1. **Root Mean Squared (RMS)**

$\mathrm{RMS}=\sqrt{\frac{1}{N_{p}}\sum_{i=1}^{N_{p}} \left( X(i)+c \right)^{2}}$

1. **Variance**

$\mathrm{variance}=\frac{1}{N_{p}}\sum_{i=1}^{N_{p}} \left( X(i)-\bar{X} \right)^{2}$

1. **Uniformity**

$\text{uniformity}=\sum_{i=1}^{N_{g}} p(i)^{2}$

1. **Median**

The median gray level intensity within the ROI。

1. **Interquartile Range**

interquartile range=P75−P25

Here P25 and P75 are the 25^th^ and 75^th^ percentile of the image array, respectively.

*Gray Level Co-occurrence Matrix (GLCM) Features*

A Gray Level Co-occurrence Matrix (GLCM) of size Ng × Ng describes the second-order joint probability function of an image region constrained by the mask and is defined as P(i,j|δ, θ). The (i, j)^tℎ^ element of this matrix represents the number of times the combination of levels i and j occur in two pixels in the image, that are separated by a distance of δ pixels along angle θ. The distance δ from the center voxel is defined as the distance according to the infinity norm. For δ = 1, this results in 2 neighbors for each of 13 angles in 3D (26-connectivity) and for δ = 2 a 98-

connectivity (49 unique angles).

Let:

**.** $\epsilon$ be an arbitrarily small positive number (≈ 2.2 × 10 ^− 16^)

**.** P (i,j) be the co-occurrence matrix for an arbitrary δ and θ

**.** p (i, j) be the normalized co-occurence matrix and equal to
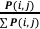


**.** N_g_ be the number of discrete intensity levels in the image

**.** p_x_ (i) = ∑
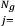
1 P(i,j) be the marginal row probabilities

**.** p_y_ (j) = ∑
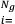
1 P(i,j) be the marginal column probabilities

**.** μ_x_ be the mean gray level intensity of p_x_ and defined as μ_x_ = ∑
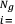
1 p_x_ (i) i.

**.** μ_y_ be the mean gray level intensity of p_y_ and defined as μ_y_ = ∑
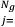
1 p_y_ (j) j.

**.** σ_x_ be the standard deviation of p_x_

**.** σ_y_ be the standard deviation of p_y_

**.** p_x+y_(k) = ∑
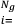
1 ∑
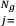
1 p(i,j), where i + j = k, and k = 2,3, … ,2N_g_ .

**.** p_x −y_ (k) = ∑
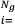
1 ∑
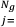
1 p(i,j), where |i − j| = k, and k = 0, 1, … , N_g_ − 1.

**.** HX = − ∑
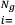
1 p_x_ (i)log_2_ (p_x_ (i) + E) be the entropy of p_x_

**.** HY = − ∑
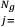
1 p_y_ (j)log_2_ (p_y_ (j) + E) be the entropy of p_y_

**.** HXY = − ∑
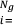
1 ∑
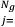
1 p(i,j) log_2_ (p(i,j) + E) be the entropy of p (i,j).

**.** HXY1 = − ∑
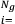
1 ∑
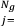
1 p(i,j) log_2_ (p_x_ (i)p_y_ (j) + $\epsilon$).

**.** HXY2 = − ∑
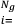
1 ∑
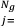
1 p_x_ (i)p_y_ (j) log_2_ (p_x_ (i)p_y_ (j) + $\epsilon)$

The following GLCM features were extracted, and a few radiomics features were selected as representative examples for presentation:

1. **Autocorrelation**

$\text{autocorrelation}=\sum_{i=1}^{N_{g}} \sum_{j=1}^{N_{g}} p(i,j)ij$

1. **Joint Average**

$\text{joint average}=\mu_{x}=\sum_{i=1}^{N_{g}} \sum_{j=1}^{N_{g}} p(i,j)i$

1. **Cluster Shade**

$\text{cluster shade}=\sum_{i=1}^{N_{g}} \sum_{j=1}^{N_{g}} {(i+j-\mu_{x}-\mu_{y})}^{3}p(i,j)$

1. **Cluster Prominence**

$\left. \text{cluster prominence}=\sum_{i=1}^{N_{g}} \sum_{j=1}^{N_{g}} \left( \begin{aligned} i+j-\mu_{x}-\mu_{y} \end{aligned} \right. \right)^{4}p(i,j)$

1. **Cluster Tendency**

$\left. \text{cluster tendency}=\sum_{i=1}^{N_{g}} \sum_{j=1}^{N_{g}} \left( \begin{aligned} i+j-\mu_{x}-\mu_{y} \end{aligned} \right. \right)^{2}p(i,j)$

1. **Correlation**

$\text{correlation}=\frac{\sum_{i=1}^{Ng} \sum_{j=1}^{Ng} p(i,j)ij-\mu_{\chi}\mu_{y}}{\sigma_{\chi}(i)\sigma_{y}(j)}$

1. **Difference Average**

$\text{difference average}=\sum_{k=0}^{N_{g}-1} kp_{x-y}(k)$

*Gray Level Run Length Matrix (GLRLM) Features*

A Gray Level Run Length Matrix (GLRLM) quantifies gray level runs, which are defined as the length in number of pixels, of consecutive pixels that have the same gray level value. In a gray level run length matrix P(i, j|θ), the (i,j)^tℎ^ element describes the number of runs with gray level

i and length j occur in the image (ROI) along angle θ.

Let:

**.** N_g_ be the number of discreet intensity values in the image

**.** N_r_ be the number of discreet run lengths in the image

**.** N_p_ be the number of voxels in the image

**.** N_r_ (θ) be the number of runs in the image along angle θ, which is equal to ∑
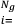
1 ∑
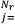
1 P(i,j|θ) and 1≤ N_r_ (θ) ≤ N_p_ .

**.** P(i,j|θ) be the run length matrix for an arbitrary direction θ .

**.** p(i, j|θ) be the normalized run length matrix, defined asp(i,j|θ) =
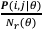


1. **Short Run Emphasis (SRE)**

$\mathrm{SRE}=\frac{\sum_{i=1}^{N_{g}} \sum_{j=1}^{N_{r}} \frac{P(i,j\mid\theta)}{j^{2}}}{N_{r}(\theta)}$

1. **Long Run Emphasis (LRE)**

$\mathrm{LRE}=\frac{\sum_{i=1}^{Ng} \sum_{j=1}^{N_{r}} P(i,j\mid\theta)j^{2}}{N_{r}(\theta)}$

1. **Gray Level Non-Uniformity (GLN)**

$$\mathrm{GLN}=\frac{\sum_{i=1}^{N_{g}} \left( \sum_{j=1}^{N_{r}} P(i,j\mid\theta) \right)^{2}}{N_{r}(\theta)}$$

2. **Gray Level Non-Uniformity Normalized (GLNN)**

$$\mathrm{GLNN}=\frac{\sum_{i=1}^{N_{g}} \left( \sum_{j=1}^{N_{r}} P(i,j\mid\theta) \right)^{2}}{N_{r}(\theta)^{2}}$$

3. **Run-Length Nonuniformity (RLN)**

$$\mathrm{RLN}=\frac{\sum_{j=1}^{N_{r}} \left( \sum_{i=1}^{N_{g}} P(i,j\mid\theta) \right)^{2}}{N_{r}(\theta)}$$

4. **Run Length Non-Uniformity Normalized (RLNN)**

$\mathrm{RLNN}=\frac{\sum_{j=1}^{N_{r}} \left( \sum_{i=1}^{N_{g}} P(i,j\mid\theta) \right)^{2}}{N_{r}(\theta)^{2}}$

1. **Run Percentage (RP)**

$\mathrm{RP}=\frac{N_{r}(\theta)}{N_{p}}$

1. **Gray-Level Variance (GLV)**

$$\mathrm{GLV}=\sum_{i=1}^{N_{g}} \sum_{j=1}^{N_{r}} p(i,j|\theta)(i-\mu)^{2}$$

2. **Run Variance (RV)**

$$\mathrm{RV}=\sum_{i=1}^{N_{g}} \sum_{j=1}^{N_{r}} p(i,j|\theta)(j-\mu)^{2}$$

Here, $\mu=\sum_{i=1}^{N_{g}} \sum_{j=1}^{N_{r}} p(i,j\mid\theta)j$

1. **Run Entropy (RE)**

$\mathrm{RE}=-\sum_{i=1}^{N_{g}} \sum_{j=1}^{N_{r}} p(i,j\mid\theta)\log_{2}(p(i,j\mid\theta)+\epsilon)$

Here, $\epsilon$ is an arbitrarily small positive number (≈2.2×10^−16^).

1. **Low Gray-Level Run Emphasis (LGLRE)**

$\mathrm{LGLRE}=\frac{\sum_{i=1}^{N_{g}} \sum_{j=1} \frac{P(i,j\mid\theta)}{i^{2}}}{N_{r}(\theta)}$

1. **High Gray-Level Run Emphasis (HGLRE)**

$$\mathrm{HGLRE}=\frac{\sum_{i=1}^{Ng} \sum_{j=1}^{N_{r}} P(i,j|\theta)i^{2}}{N_{r}(\theta)}$$

2. **Short Run Low Gray-Level Emphasis (SRLGE)**

$$\mathrm{SRLGLE}=\frac{\sum_{i=1}^{N_{g}} \sum_{j=1}^{N_{r}} \frac{P(i,j\mid\theta)}{i^{2}j^{2}}}{N_{r}(\theta)}$$

3. **Short Run High Gray-Level Emphasis (SRHGE)**

$$\mathrm{SRHGLE}=\frac{\sum_{i=1}^{N_{g}} \sum_{j=1}^{N_{r}} \frac{P(i,j\mid\theta)i^{2}}{j^{2}}}{N_{r}(\theta)}$$

4. **Long Run Low Gray-Level Emphasis (LRLGE)**

$$\mathrm{LRLGLRE}=\frac{\sum_{i=1}^{Ng} \sum_{j=1}^{N_{r}} \frac{P(i,j\mid\theta)j^{2}}{i^{2}}}{N_{r}(\theta)}$$

5. **Long Run High Gray-Level Emphasis (LRHGE)**

$$\mathrm{LRHGLRE}=\frac{\sum_{i=1}^{N_{g}} \sum_{j=1}^{N_{r}} P(i,j\mid\theta)i^{2}j^{2}}{N_{r}(\theta)}$$

*Gray Level Size Zone Matrix (GLSZM) Features*

A Gray Level Size Zone (GLSZM) quantifies gray level zones in an image. A gray level zone is defined as a number of connected voxels that share the same gray level intensity. A voxel is considered connected if the distance is 1 according to the infinity norm (26-connected region in a 3D, 8-connected region in 2D). In a gray level size zone matrix P(i,j) the (i,j)th element equals the number of zones with gray level i and size j appear in image. Contrary to GLCM and GLRLM, the GLSZM is rotation independent, with only one matrix calculated for all directions in the ROI.

Let:

**.** N_g_ be the number of discreet intensity values in the image

**.** N_s_ be the number of discreet zone sizes in the image

**.** N_p_ be the number of voxels in the image

**.** N_z_ be the number of zones in the ROI, which is equal to ∑
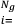
1 ∑
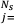
1 P(i,j) and 1 ≤ N_z_ ≤ N_p_

**.** P(i,j) be the size zone matrix

**.** p(i, j) be the normalized size zone matrix, defined as p(i,j) =
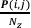


We extracted several representative GLSZM features are as follows:

1. **Small Area Emphasis (SAE)**

$\mathrm{SAE}=\frac{\sum_{i=1}^{N_{g}} \sum_{j=1}^{N_{s}} \frac{P(i,j)}{j^{2}}}{N_{z}}$

1. **Large Area Emphasis (LAE)**

$\mathrm{LAE}=\frac{\sum_{i=1}^{Ng} \sum_{j=1}^{N_{s}} P(i,j)j^{2}}{N_{z}}$

1. **Gray Level Non-Uniformity (GLN)**

$\mathrm{GLN}=\frac{\sum_{i=1}^{N_{g}} \left( \sum_{j=1}^{N_{s}} P(i,j) \right)^{2}}{N_{z}}$

1. **Gray Level Non-Uniformity Normalized (GLNN)**

$\mathrm{GLNN}=\frac{\sum_{i=1}^{N_{g}} \left( \sum_{j=1}^{N_{s}} P(i,j) \right)^{2}}{N_{z}^{2}}$

1. **Size-Zone Non-Uniformity (SZN)**

$\mathrm{SZN}=\frac{\sum_{j=1}^{N_{S}} \left( \sum_{i=1}^{N_{g}} P(i,j) \right)^{2}}{N_{z}}$

1. **Size-Zone Non-Uniformity Normalized (SZNN)**

$$\mathrm{SZNN}=\frac{\sum_{j=1}^{N_{S}} \left( \sum_{i=1}^{N_{g}} P(i,j) \right)^{2}}{N_{z}^{2}}$$

*Gray Level Dependence Matrix (GLDM) Features*

A Gray Level Dependence Matrix (GLDM) quantifies gray level dependencies in an image. A gray level dependency is defined as the number of connected voxels within distance δ that are dependent on the center voxel. A neighboring voxel with gray level j is considered dependent on center voxel with gray level i if |i − j| ≤ a. In a gray level dependence matrix P(i,j) the (i,j)tℎ element describes the number of times a voxel with gray level i with j dependent voxels in its

neighborhood appears in image.

Let:

**.** N_g_ be the number of discreet intensity values in the image

**.** N_d_ be the number of discreet dependency sizes in the image

**.** N_z_ be the number of dependency zones in the image, which is equal to∑
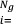
1 ∑
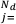
1 P(i,j)

**.** P(i,j) be the dependence matrix

**.** p(i,j) be the normalized dependence matrix, defined as p(i,j) =
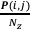


We extracted several representative GLDM features are as follows:

1. **Small Dependence Emphasis (SDE)**

$SDE=\frac{\sum_{i=1}^{N_{g}} \sum_{j=1}^{N_{d}} \frac{P(i,j)}{i^{2}}}{N_{z}}$

1. **Large Dependence Emphasis (LDE)**

$$LDE=\frac{\sum_{i=1}^{Ng} \sum_{j=1}^{N_{d}} P(i,j)j^{2}}{N_{z}}$$

2. **Gray Level Non-Uniformity (GLN)**

$$GLN=\frac{\sum_{i=1}^{N_{g}} \left( \sum_{j=1}^{N_{d}} P(i,j) \right)^{2}}{N_{z}}$$

3. **Dependence Non-Uniformity (DN)**

$$DN=\frac{\sum_{j=1}^{N_{d}} \left( \sum_{i=1}^{N_{g}} P(i,j) \right)^{2}}{N_{z}}$$

4. **Dependence Non-Uniformity Normalized (DNN)**

$$DNN=\frac{\sum_{j=1}^{N_{d}} \left( \sum_{i=1}^{N_{g}} P(i,j) \right)^{2}}{N_{z}^{2}}$$

*Neighbouring Gray Tone Difference Matrix (NGTDM) Features*

A Neighboring Gray Tone Difference Matrix quantifies the difference between a gray value and the average gray value of its neighbors within distance δ. The sum of absolute differences for gray level i is stored in the matrix. Let xgl be a set of segmented voxels and xgl (jx,jy,jz ) ∈ xgl be the gray level of a voxel at position (jx,jy,jz ), then the average gray level of the neigbourhood is:

$$\begin{aligned} \bar{A}_{i}=\bar{A}(j_{x},j_{y},j_{z}) \\ =\frac{1}{W}\sum_{k_{x}=-\delta}^{\delta} \sum_{k_{y}=-\delta}^{\delta} \sum_{k_{z}=-\delta}^{\delta} x_{gl}(j_{x}+k_{x},j_{y}+k_{y},j_{z}+k_{z}), \\ \mathrm{where}(k_{x},k_{y},k_{z})\neq(0,0,0)\mathrm{and}\chi_{gl}(j_{x}+k_{x},j_{y}+k_{y},j_{z}+k_{z})\in X_{gl} \end{aligned}$$

Here, *W* is the number of voxels in the neighbourhood that are also in xgl.

Let:

**.** n*_i_* be the number of voxels in xgl with gray level *i*.

**.** N*_v,p_* be the total number of voxels in x*_gl_* and equal to ∑n*_i_* (i.e., the number of voxels with a valid region; at least 1 neighbor). N*_v,p_* ≤ N*_p_*, where N*p* is the total number of voxels in the ROI.

**.** p*_i_* be the gray level probability and equal to n*_i_*/N*_v._*

$\left. s_{i}=\left\{ \begin{matrix} \Sigma^{n_{i}} & \mid i-\bar{A}_{i}\mid& \text{for} & n_{i}\neq0 \\ 0 & \text{for} & n_{i}=0 & \end{matrix} \right. \right.$ be the sum of absolute differences for gray level *i*.

**.** N*_g_* be the number of discreet gray levels.

**.** N*_g,p_* be the number of gray levels where p*_i_* ≠ 0.

1. **Coarseness**

$$Coarseness=\frac{1}{\sum_{i=1}^{N_{g}} p_{i}s_{i}}$$

2. **Contrast**

$$Contrast=\left( \frac{1}{N_{g,p}\left( N_{g,p}-1 \right)}\sum_{i=1}^{N_{g}} \sum_{j=1}^{N_{g}} p_{i}p_{j}(i-j)^{2} \right)\left( \frac{1}{N_{v,p}}\sum_{i=1}^{N_{g}} s_{i} \right),$$

$$\text{where }p_{i}\neq0,p_{j}\neq0$$

3. **Busyness**

$$Busyness=\frac{\sum_{i=1}^{N_{g}} p_{i}s_{i}}{\sum_{i=1}^{N_{g}} \sum_{j=1}^{N_{g}} \mid ip_{i}-jp_{j}\mid}\text{,where}p_{i}\neq0,p_{j}\neq0$$

4. **Complexity**

$Complexity=\frac{1}{N_{v,p}}\sum_{i=1}^{N_{g}} \sum_{j=1}^{N_{g}} \mid i-j\mid\frac{p_{i}s_{i}+p_{j}s_{j}}{p_{i}+p_{j}},\mathrm{where}p_{i}\neq0,p_{j}\neq0$

1. **Strength**

$$Complexity=\frac{1}{N_{v,p}}\sum_{i=1}^{N_{g}} \sum_{j=1}^{N_{g}} \mid i-j\mid\frac{p_{i}s_{i}+p_{j}s_{j}}{p_{i}+p_{j}},\mathrm{where}p_{i}\neq0,p_{j}\neq0$$

*Shape Features (3D)*

In this group of features we included descriptors of the three-dimensional size and shape of the ROI. These features are independent from the gray level intensity distribution in the ROI and are therefore only calculated on the non-derived image and mask.

Let:

- N*_v_* represent the number of voxels included in the ROI
- N*_f_* represent the number of faces (triangles) defining the Mesh.
- V the volume of the mesh in mm^3^, calculated by [getMeshVolumeFeatureValue()](https://pyradiomics.readthedocs.io/en/latest/features.html#radiomics.shape.RadiomicsShape.getMeshVolumeFeatureValue)
- A the surface area of the mesh in mm^2^, calculated by getMeshSurfaceAreaFeatureValue()

We extracted several representative shape features are as follows:

1. **Elongation**

$$\text{elongation}=\sqrt{\frac{\lambda_{minor}}{\lambda_{major}}}$$

2. **Mesh Volume**

$$\begin{aligned} V_{i}=\frac{Oa_{i}\cdot(Ob_{i}\times Oc_{i})}{6}\left( 1 \right) \\ V=\sum_{i=1}^{Nf} V_{i}\left( 2 \right) \end{aligned}$$

3. **Voxel Volume**

$V_{voxel}=\sum_{k=1}^{N_{v}} V_{k}$

1. **Surface Area**

$\begin{aligned} A_{i}&=\frac{1}{2}\mid a_{i}b_{i}\times a_{i}c_{i}\mid(1) \\ A&=\sum_{i=1}^{N_{f}} A_{i}\left( 2 \right) \end{aligned}$

1. **Sphericity**

$\text{sphericity}=\frac{\sqrt[3]{36\pi V^{2}}}{A}$

Reference:

1. van Griethuysen JJM, Fedorov A, Parmar C, et al. Computational radiomics system to decode the radiographic phenotype. Cancer Res. 2017;77(21):e104–e107. doi: 10.1158/0008-5472.CAN-17-0339.

**Supplementary Methods 3.**

**Radiomics score calculation formula:**

Radiomics score = -1.49177774 - 0.64425768 × original_glszm_SmallAreaLowGrayLevelEmphasis

- 1.05391969 × wavelet-LLH_ngtdm_Strength

+ 0.152321293 × wavelet-LHH_glcm_SumSquares

+ 0.49892000 × wavelet-LHH_glszm_SizeZoneNonUniformity

+ 0.05742943 × wavelet-HLL_gldm_DependenceVariance

+ 0.64928466 × wavelet-HLH_glszm_SizeZoneNonUniformity

+ 0.44268417 × wavelet-HLH_glszm_SmallAreaHighGrayLevelEmphasis

+ 0.36779878 × wavelet-HHL_firstorder_RootMeanSquared

+ 0.67870749 × wavelet-HHH_firstorder_Mean

+ 0.44691110 × wavelet-HHH_glszm_SizeZoneNonUniformity

- 0.26703662 × wavelet-LLL_gldm_LowGrayLevelEmphasis

-0.52299494 × wavelet-LLL_gldm_SmallDependenceLowGrayLevelEmphasis

**Supplementary Methods 4.**


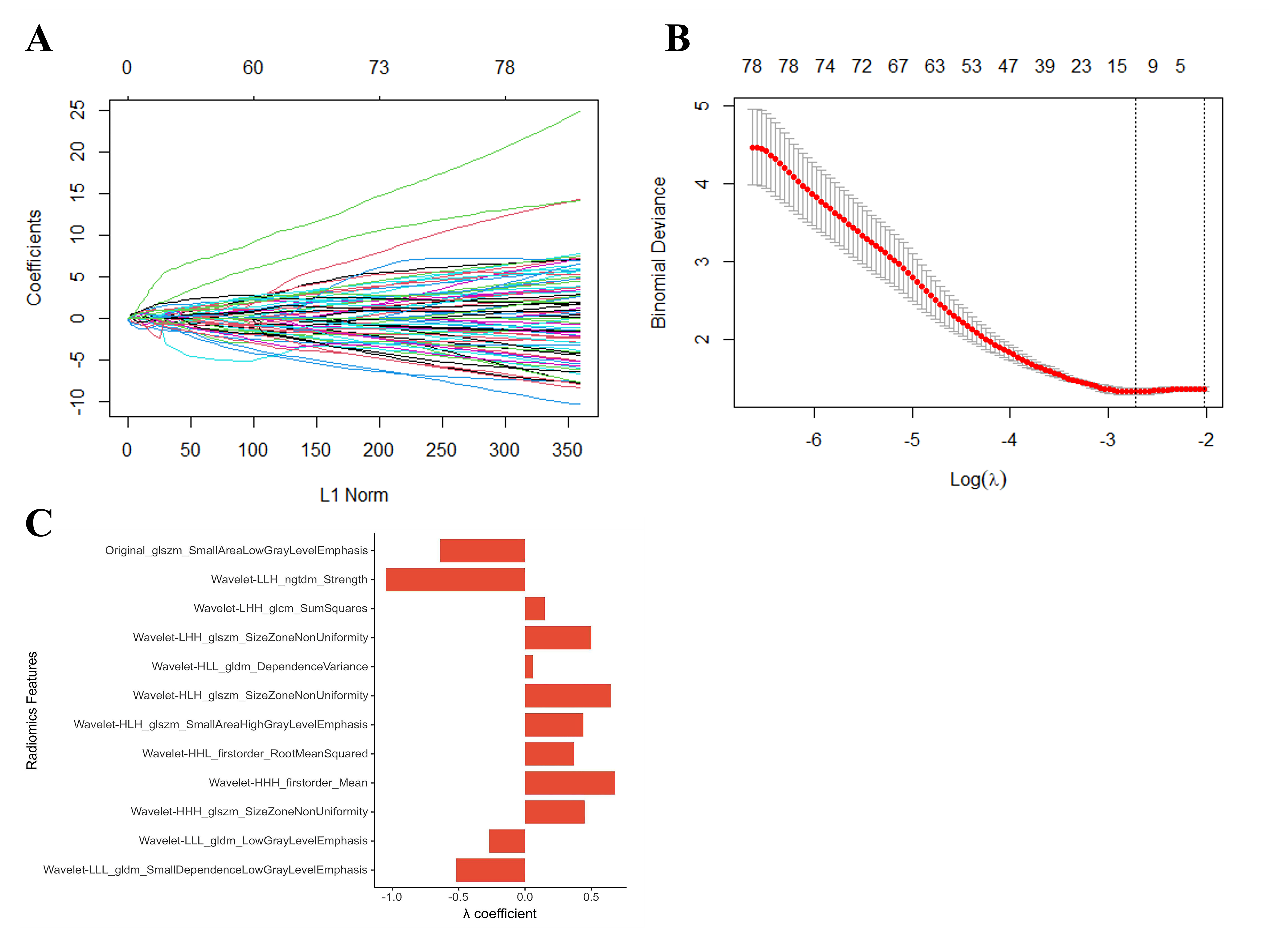


Figure S1. Radiomics feature selection was conducted using least absolute shrinkage and selection operator (LASSO) logistic regression to establish the radiomics score (A-C). In analysis, twelve radiomics features were selected by identifying the best λ in the LASSO model (A, B), and their weights were listed in (C). Glszm, gray-level size zone matrix; LLH, low-low-high-pass filtered image; LHH, low-high-high-pass filtered image; HLL high-low-low-pass filtered image; HLH = high-low-high-pass filtered image; HHH, high-high-high-pass filtered image; LLL, low-low-low-pass filtered image.
